# Supplementary material for: Designing an App to Promote Physical Exercise in Sedentary People Using a Day-to-Day Algorithm to Ensure a Healthy Self-Programmed Exercise Training
Source: Int J Environ Res Public Health. 2023 Jan 14;20(2):1528. doi: 10.3390/ijerph20021528 (PMC9861270; doi:10.3390/ijerph20021528)
Supplement: Supplementary file 1 [file ijerph-20-01528-s001.zip › ijerph-2082068-supplementary.pdf]

ELECTRONIC SUPPLEMENTARY MATERIAL

Section S1: Physical exercise programme from the Selftraining UMH app.

The training programme consists of 3 sessions a week for 3 months. It is an individualised programme through heart rate variability (HRV), focused on over 18-year-old people who do not practice physical activity and want to start leading a healthier lifestyle.

During the sessions, a combination of strength and resistance exercises are performed using materials that can be found at home (water bottles, books, backpacks, broomsticks, chairs...). The sessions increase in intensity and duration as participants improve their physical condition.

The sessions consist of a general warm-up, a specific warm-up, the main part, and a cool-down.

In the first two weeks there are 3 low intensity sessions of adaptation to the training and correction of errors for a good technical performance of the exercises. From week 3 onwards, 2 high-intensity sessions plus one low-intensity session are carried out to complete the 3 weekly sessions, using the decision-making algorithm used by Javaloyes et al. (2019) which is a modification of Kiviniemi et al. (2007). The protocol is as follows.

Level 1 (low intensity): Conventional strength exercises.

4-5 sets of 4 exercises each set, in which 15 repetitions of each exercise are performed, with 1 minute rest between exercises and 2 minutes rest between sets.

The exercises to be performed are squat, lunges, push-ups, vertical press, rowing, thruster, front plank, side plank, dorsal bridge (static/dynamic), dead bag and bird dog.

Level 2 (high intensity): High-intensity interval training is performed with a combination of strength and endurance exercises.

- 2 sets of 10-12 exercises each set, with 30 seconds of work and 30 seconds of rest, with a 2-minute rest between sets.

Level 3 (high intensity): High intensity interval training is performed with a combination of strength and endurance exercises.

- 3 sets of 8-10 exercises each set, with 30 seconds of work and 30 seconds of rest, with a 2-minute rest between sets.

Level 4 (high intensity): High intensity interval training is performed with a combination of strength and endurance exercises.

- 3 sets of 8-10 exercises each set, with 40 seconds of work and 20 seconds of rest, with a 2-minute rest between sets.

The exercises to be performed are those of level 1 combined with skipping, side steps, jumping jacks, climber, boxing, burpees...
